# Smoking, environmental tobacco smoke and occupational irritants increase the risk of chronic rhinitis

**DOI:** 10.1186/s40413-018-0184-5

**Published:** 2018-03-14

**Authors:** Hanna Hisinger-Mölkänen, Päivi Piirilä, Tari Haahtela, Anssi Sovijärvi, Paula Pallasaho

**Affiliations:** 1 Heart and Lung Center, Helsinki University Hospital, Helsinki, Finland; 2 HUS Medical Imaging Center, Helsinki University Hospital, Helsinki, Finland; 3 Skin and Allergy Hospital, Helsinki University Hospital, University of Helsinki, Helsinki, Finland; 4 HUS Medical Imaging Center, Helsinki University Hospital, and University of Helsinki, Helsinki, Finland; 5 Espoo City Health Services, Helsinki, Finland

**Keywords:** Allergic rhinitis, Environmental tobacco smoke, Occupational exposure, Rhinitis, Smoking

## Abstract

**Background:**

Allergic and non-allergic rhinitis cause a lot of symptoms in everyday life. To decrease the burden more information of the preventable risk factors is needed. We assessed prevalence and risk factors for chronic nasal symptoms, exploring the effects of smoking, environmental tobacco smoke, exposure to occupational irritants, and their combinations.

**Methods:**

In 2016, a postal survey was conducted among a random population sample of 8000 adults in Helsinki, Finland with a 50.5% response rate.

**Results:**

Smoking was associated with a significant increase in occurrence of chronic rhinitis (longstanding nasal congestion or runny nose), but not with self-reported or physician diagnosed allergic rhinitis. The highest prevalence estimates of nasal symptoms, 55.1% for chronic rhinitis, 49.1% for nasal congestion, and 40.7% for runny nose, were found among smokers with occupational exposure to gases, fumes or dusts.

Besides active smoking, also exposure to environmental tobacco smoke combined with occupational exposure increased the risk of nasal symptoms.

**Conclusions:**

Smoking, environmental tobacco smoke, and occupational irritants are significant risk factors for nasal symptoms with an additive pattern. The findings suggest that these factors should be systematically inquired in patients with nasal symptoms for appropriate preventive measures. (192 words).

## Background

It is estimated that 500 million people worldwide suffer from allergic rhinitis and in addition 200 million from non-allergic rhinitis [1, 2]]. These conditions render a considerable burden on both the patients and society in terms of life quality, disability and costs [3–5].

Allergic rhinitis is considered to be part of a systemic allergic condition often with comorbidities [6]. Rhinitis is a risk factor for asthma and eczema [7–9], and affects asthma and COPD control [10, 11]. Non-allergic rhinitis is a heterogenous group of conditions with variable etiology, e.g. hormonal, drug-induced, irritatative or idiopathic [2].

Rhinitis symptoms and chronic rhinosinusitis are significantly more common among smoking individuals than among non-smokers [12] . Smoking is also associated with the development of nasal polyposis [13]. The harmful effects of environmental tobacco smoke are also known from previous studies [14–17]. Occupational exposure to gases, dusts or fumes is a risk factor for rhinitis and sinusitis [16, 18] as well as for asthma and COPD [19], and eczema [9] . The combined effects of smoking, environmental tobacco smoke and occupational irritants on nasal symptoms have been less well estimated.

The FinEsS study is a Nordic joint project on the occurrence and risk factors of respiratory conditions in Finland, Estonia and Sweden. The present study assessed the prevalence of nasal symptoms and their association with smoking, environmental tobacco smoke, and occupational irritants in a population based cohort of 8000 Finnish adults.

## Methods

### Study population

In 2016, a postal questionnaire was mailed to a randomly selected sample of 8000 adults aged 20–69 years in 10-year age cohorts corresponding the gender and age distribution of the Finnish population. The genders were randomized separately. The sample was obtained from the Finnish National Population Registry (permission Dnro 254/410/15; 8.1.2015).

The invited individuals were given the possibility to respond either by mail or on the internet. Reminders were sent twice. The questionnaire was mailed in Finnish, Swedish or English, depending on the individuals` language. Of the 8000 selected, 17 refused to participate, 7 mailed an empty questionnaire and one had died. Of the 7975 invited 4026 (50.5%) responded. Twenty-eight questionnaires were excluded because they were not adequately fulfilled. For the present analysis, 510 individuals of the whole study population were excluded, because the questions about smoking habits, environmental tobacco smoke or exposure to gases, dusts or fumes at work were not answered. Thus, the final sample included 3488 individuals (1997, 57.3% women; 1491, 42.7% men).

The study was approved by the Coordinating Ethics Committee of Helsinki and Uusimaa Hospital District (200/13/03/00/2015).

### Questionnaire

In Helsinki, previous postal surveys were conducted in 1996 and 2006 [17]. In 2016 we used the same questionnaire as in 1996 and 2006, but also additional questions about occupational and environmental exposure, nasal symptoms and allergy were included.

### Questions and definitions

*Chronic rhinitis*. A positive answer to either having longstanding nasal congestion or runny nose or both.

*Nasal congestion.* Have you had longstanding nasal congestion?

*Runny nose.* Have you had longstanding rhinitis?

*Self-reported allergic rhinitis*. Do you have now or have you had previously allergic rhinitis (e.g. hay fever) or allergic eye symptoms?

*Physician diagnosed allergic rhinitis*. Have you been diagnosed by a doctor as having allergic rhinitis?

*Smoker*. Current smoker or having stopped smoking during the last year.

*Ex-smoker*. Stopped smoking more than 12 months prior to the study.

*Non-smoker*. Neither current smoker nor ex-smoker.

*Exposure to tobacco smoke at work*. Are you now or have you been heavily exposed to tobacco smoke at work?

*Exposure to tobacco smoke at home*. Are you now or have you been heavily exposed to tobacco smoke at home?

*Exposure to environmental tobacco smoke*. A positive answer to either exposure to tobacco smoke at work or at home.

*Exposure to gases, dusts or fumes at work* (*occupational irritants*). Are you now or have you been heavily exposed to gases, dusts or fumes at work?

### Statistical analysis

All analyses were performed using SPSS version 23.0. Comparisons of proportions were tested with the Mann-Whitney U-test. *p* < 0.05 was regarded as statistically significant. Multiple logistic regression models were performed using smoking, exposure to tobacco smoke, and exposure to gases, dusts or fumes at work as risk factors for chronic nasal symptoms. Odds ratios (ORs) are presented with 95% confidence intervals (95%CI).

## Results

The characteristics of the study population are shown in Table 1. In total, 26.6% of the responders were smokers, and 26% ex-smokers. Smoking was more common among younger individuals, 31.6% of the responders aged 20–29 years were smokers compared to 21.4% of those aged 60–69 years (*p* = 0.00). Exposure to gases, dusts or fumes was reported by 26.8% of the responders. Exposure to these irritants was slightly less frequent among younger individuals, 24.7% of those aged 20–29 years had been exposed compared to 28.6% of those aged 60–69 years (*p* = 0.068).

**Table 1:** Age, gender, smoking, environmental tobacco smoke and occupational irritants of the studied population

	All	Men	Women
*N* (%)	3488	1491(42.7%)	1997(57.3%)
Mean (SD) age, years	45.0(14.7)	45.3(14.4)	44.9(14.9)
Age 20–29	643(18.4%)	246(16.5%)	397(19.9%)
30–39	785(22.5%)	361(24.2%)	424(21.2%)
40–49	564(16.2%)	249(16.7%)	315(15.8%)
50–59	712(20.4%)	305(20.5%)	407(20.4%)
60–69	740(21.2%)	314(21.1%)	426(21.3%)
Smokers	929 (26.6%)	464(31.1%)	465(23.3%)
Ex-smokers	907(26.0%)	425(28.5%)	482(24.1%)
Non-smokers	1764(50.6%)	659(44.2%)	1105(55.3%)
Exposure to tobacco smoke at work	326(9.3%)	169(11.3%)	157(7.9%)
Exposure to tobacco smoke at home	358(10.3%)	170(11.4%)	188(9.4%)
Exposure to occupational irritants	934(26.8%)	479(32.1%)	455(22.8%)

Age, gender, smoking and exposure characteristics of the studied population. Occupational irritants = exposure to gases, dust or fumes at work. The figures indicate the number of subjects except age groups indicated in years

**Fig. 1:**
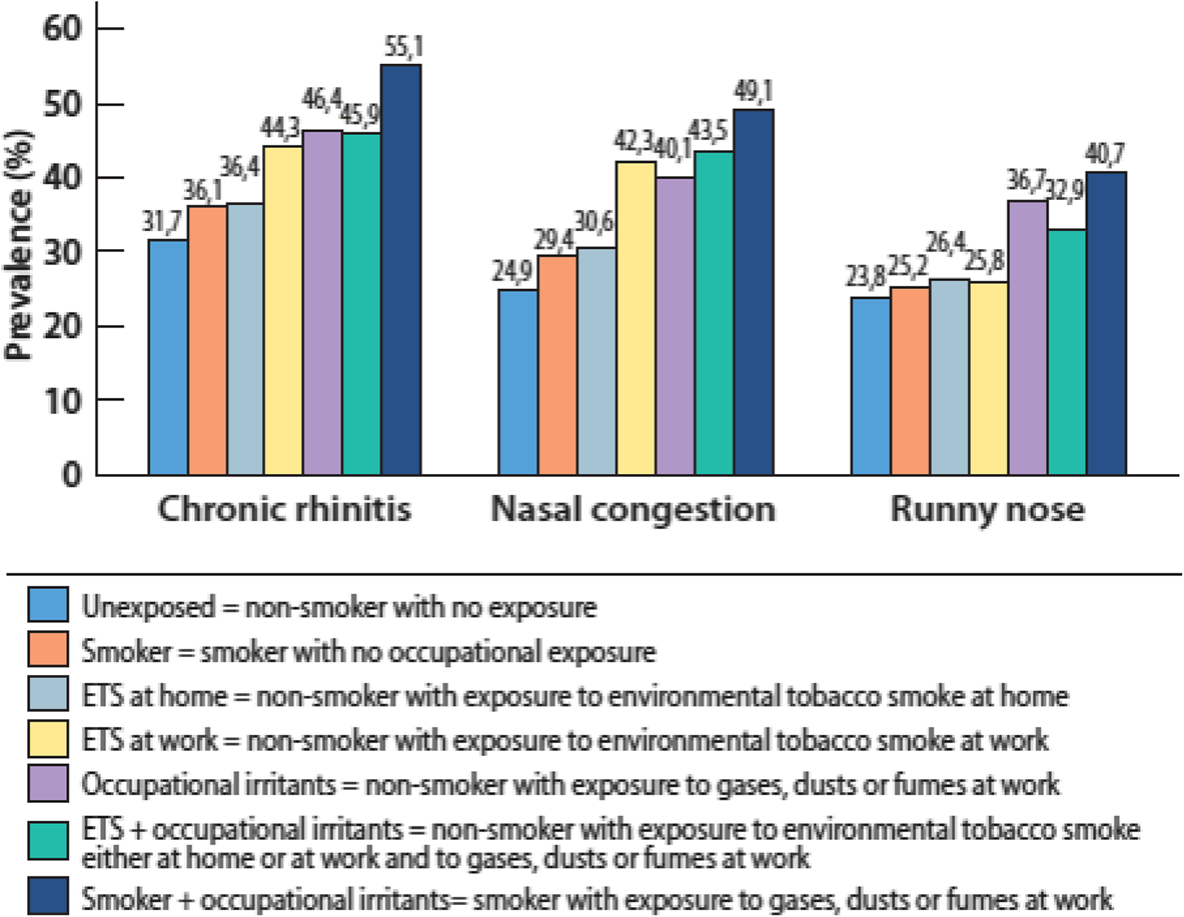
Prevalence of chronic rhinitis, nasal congestion and runny nose in different exposures and their combinations

In the whole study sample, prevalence of chronic rhinitis (longstanding nasal congestion or runny nose) was 36.9%, of nasal congestion 29.8%, and of runny nose 27.9%. Smoking, environmental tobacco smoke and occupational irritants increased the prevalence in an additive manner (Fig. 1, Table 2). Smoking alone increased slightly the nasal symptoms, but was not associated with self-reported allergic rhinitis or physician-diagnosed allergic rhinitis. However, if a smoker was also exposed to occupational irritants, the prevalence of nasal symptoms was the highest. This additive effect of exposure to tobacco smoke and occupational irritants on chronic nasal symptoms remained when data were stratified by physician diagnosed allergic rhinitis (Table 2).

**Table 2:** Prevalence of chronic rhinitis, nasal congestion and runny nose

	Unexposed	Smoker	ETS at home	ETS at work	Occupatio-nal irritants	ETS + Occupational irritants	Smoker + Occupational irritants
All responders							
Chronic rhinitis	31.7%	36.1% 0.073	36.4% 0.29	44.3% 0.01	46.4% 0.000	45.9% 0.007	55.1% 0.000
Nasal congestion	24.9%	29.4% 0.049	30.6% 0.17	42.3% 0.000	40.1% 0.000	43.5% 0.000	49.1% 0.000
Runny nose	23.8%	25.2% 0.54	26.4% 0.51	25.8% 0.65	36.7% 0.000	32.9% 0.056	40.7% 0.000
Responders with physician diagnosed allergic rhinitis							
Chronic rhinitis	51.4%	50.0% 0.77	54.1% 0.76	70.0% 0.051	66.7% 0.006	65.5% 0.14	69.6% 0.002
Nasal congestion	42.9%	43.0% 0.98	51.4% 0.33	66.7% 0.012	57.1% 0.010	65.5% 0.019	64.1% 0.000
Runny nose	40.1%	39.2% 0.86	40.5% 0.96	46.7% 0.48	59.0% 0.001	48.3% 0.39	53.3% 0.023
Responders without physician diagnosed allergic rhinitis							
Chronic rhinitis	24.3%	31.1% 0.008	28.6% 0.39	32.8% 0.12	37.7% 0.000	35.7% 0.056	49.6% 0.000
Nasal congestion	18.2%	24.5% 0.007	21.4% 0.47	31.3% 0.008	32.8% 0.000	32.1% 0.010	43.4% 0.000
Runny nose	17.7%	20.1% 0.28	20.2% 0.56	16.4% 0.79	27.0% 0.001	25.0% 0.17	36.0% 0.000

Prevalence of chronic rhinitis, nasal congestion and runny nose stratified according to risk factors: smoking, environmental tobacco smoke (ETS), occupational irritants, or their combinations among all responders and among responders with and without physician diagnosed allergic rhinitis. Probability figures are indicated below each prevalence figure. *p*-values are given between unexposed and those, who are exposed to one or more of the risk factors

The prevalence of physician diagnosed allergic rhinitis was 27.9% in the whole study sample. It was reported by 26.9% of smokers, by 28.9% of ex-smokers and by 27.8% of non-smokers (*p* = 0.072). In men, environmental tobacco smoke or occupational irritants did not associate with physician diagnosed allergic rhinitis. Among women, however, 33.7% of those exposed to occupational irritants had physician diagnosed allergic rhinitis compared with 26.1% among the non-exposed (*p* = 0.032). Also in women, if occupational exposure was combined with environmental tobacco exposure the prevalence increased from 26.1% in non-exposed to 41.3% among exposed (*p* = 0.024).

The prevalence of longstanding nasal symptoms was high also in those responders who did not report physician diagnosed allergic rhinitis, as 30.8% of them reported chronic rhinitis, 25% nasal congestion, and 21.4% runny nose.

Results of the multiple logistic regression analysis are given in Table 3. Current smoking, without exposure to occupational irritants, yielded an odds ratio (OR) 1.22 (95%CI 1.00–1.49) for chronic rhinitis, and 1.26 (95%CI 1.01–1.56) for nasal congestion. Exposure to environmental tobacco smoke at home or at work did not, as such, increase the risk significantly in our model due to collinearity.

**Table 3:** Risk factors for long-term nasal symptoms in a multiple logistic regression model

	Chronic rhinitis	Nasal congestion	Runny nose	Physician diagnosed allergic rhinitis
Unexposed	1	1	1	1
Smoker	1.22(1.00–1.49)	1.26(1.01–1.56)	1.08(0.86–1.36)	0.98(0.78–1.21)
ETS at home	0.75(0.49–1.14)	0.70(0.45–1.09)	0.75(0.47–1.21)	1.09(0.90–1.33)
ETS at work	0.99(0.58–1.73)	1.12(0.64–1.95)	0.57(0.30–1.09)	1.00(0.64–1.56)
Occupational irritants	1.28(0.97–1.69)	1.20(0.90–1.60)	1.49(1.11–1.99)	1.00(0.74–1.36)
ETS + occupational irritants	1.11(0.57–2.16)	1.31(0.66–2.58)	1.34(0.64–2.81)	1.37(0.67–2.78)
Smoker + occupational irritants	1.80(1.39–2.33)	1.82(1.40–2.35)	1.68(1.28–2.19)	0.94(0.71–1.25)

The figures indicate odds ratios (CI 95%). *ETS* Environmental tobacco smoke

Occupational irritants were associated almost significantly with chronic rhinitis OR 1.28 (95%CI 0.97–1.69), with nasal congestion OR 1.20 (95%CI 0.90–1.60), and significantly with runny nose OR 1.49 (95%CI 1.11–1.99). The risk was not further increased, if the responders were also exposed to environmental tobacco smoke.

Current smoking combined with exposure to occupational irritants gave the highest risk estimates for nasal symptoms: OR 1.80 (95%CI 1.39–2.33) for chronic rhinitis, 1.82 (95%CI 1.40–2.35) for nasal congestion, and 1.68 (95%CI 1.28–2.19) for runny nose.

## Discussion

Current smoking in combination with occupational exposure to gases, dusts or fumes resulted in increased occurrence of chronic nasal symptoms in both genders suggesting an additive harmful effect. Smoking alone was associated with chronic rhinitis and nasal congestion. Occupational irritants were associated with runny nose in both genders, and among women also with chronic rhinitis and nasal congestion. The risk increase is modest but obviously becomes significant in large populations.

The prevalence of longstanding nasal symptoms was high: 36.9% reported chronic rhinitis, 29.8% nasal congestion, and 27.9% runny nose. These symptoms were even more common than previously reported in Sweden [20, 21], but comparable to recent studies from Finland and Sweden [14, 18] .

We found exposure to occupational irritants to be a significant risk factor for chronic nasal symptoms, which is consistent with previous observations [20, 22, 23]. However, in a recent Swedish study occupational exposure to gases, dust or fumes was not associated with current rhinitis [18]. As their definition of current rhinitis included individuals with allergic rhinitis or chronic nasal symptoms, the results are not fully comparable.

Chronic nasal symptoms were more frequent among smokers and ex-smokers in the whole study group as found also in Sweden [20, 21] In the earlier report from FinEsS studies from 1997 to 2003, smoking was not associated with rhinitis symptoms [14]. In that report, however, the definition of rhinitis symptoms included also individuals with self-reported allergic rhinitis, which may explain the difference. In a study from Vietnam, using the same FinEsS-questionnaire, smoking was not a risk factor for nasal symptoms [24]. The results may be confounded by the much higher exposure to air pollution in Vietnam.

In our study, the prevalence of physician diagnosed allergic rhinitis was the same among non-smokers (27.1%) compared to current smokers (26.6%). Current smoking did not either increase the prevalence of nasal symptoms in those with physician diagnosed allergic rhinitis. However, smoking was associated with chronic nasal symptoms in the whole study sample, and increased the risk of nasal congestion significantly among those without physician diagnosed allergic rhinitis (*p* = 0.007). Our findings are in line with previous findings from U.S. [25] and suggest that the association between nasal symptoms and tobacco exposure might be independent of allergy.

The possible association between tobacco smoke and allergy has been studied previously. Smoking has been associated with an increased risk of allergic disease [26, 27] There is also evidence that tobacco smoke exposure would prevent from allergic sensitization [25, 28]. In a study from The Netherlands Vonk et al. [29] found that prenatal smoke exposure was associated with a decreased risk for the development of atopy. In another study from Canada Hancox et al. present that personal and parental smoking is associated with a lower risk of allergic sensitization in people with a family history of atopy [30]. In our study the prevalence of allergic rhinitis was independent of the smoking status.

Environmental tobacco smoke is known to be a modest risk factor for chronic nasal symptoms. Our results are in accordance with earlier findings [14] This association has also been confirmed in children [31]. We observed that environmental tobacco smoke both at home and at work slightly increased the occurrence of chronic nasal symptoms, which is in line with previous observations [32]. Environmental tobacco smoke has also predisposed to sinusitis [16].

In our study, exposure to environmental tobacco smoke at home did not increase the prevalence of chronic nasal symptoms in responders with or without physician diagnosed allergic rhinitis. Our findings are in line with previous results from Estonia; Larsson et al. found no significant association between ETS exposure at home and respiratory symptoms [33]. It is likely that our study group includes individuals that have been exposed to ETS at home in the past as smoking inside is not as common anymore as it has been in previous decades. Family members may also quit or reduce smoking inside the home more easily if one in their family develops respiratory symptoms. The lack of association might at least partly be explained by these mechanisms.

Responders who have been exposed to environmental tobacco smoke at work reported to have nasal congestion significantly more often in both groups. However, second hand smoke neither at home nor at work was a significant risk factor in our logistic regression model. Our results are in agreement with a study from Switzerland showing that second hand smoke was not independently associated with rhinitis symptoms [33].

We have found earlier, that smoking and occupational irritants have an additive effect on the incidence of COPD [19]. In the present study, we suggest that this additive exposure is also a risk factor for chronic nasal symptoms. The combined effect was seen in all age groups and whether or not allergic rhinitis was present.

Non-allergic rhinitis is divided into several subgroups. Half of the patients with non-allergic rhinitis does not have a clear etiology for their symptoms (e.g. occupational, hormonal, drug-induced) and are sometimes classified as having an idiopathic rhinitis [2]. Our results suggest that these symptoms are, at least partly, explained by smoking, environmental tobacco smoke or exposure to occupational irritants. These risks are preventable by anti-smoking efforts and reduction of occupational exposure.

### Study limitations

The observations are derived from a questionnaire survey based on a random population sample with a participation rate of 51%. With two reminders, we find the representativeness satisfactory. Participation rates have decreased by time in epidemiological studies which is a general problem. The response rate has been about the same in other epidemiological studies as in our study, for example 55% in Copenhagen City Heart Study. [34] and 53% in a Swedish follow up study on the prevalence of asthma [35].

The FinEsS questionnaire has been used in several other studies. Some new questions of nasal symptoms were added, which improved the detection of the nasal conditions. An obvious limitation of the present survey is the lack of individual clinical evaluation of the responders. We did not take into account ambient air pollution, which could potentially confound our findings. However, in Finland air pollution is minimal and well controlled, even in cities.

## Conclusions

In conclusion, smoking, environmental tobacco smoke and occupational exposure to gases, dusts or fumes increase significantly the prevalence of chronic nasal symptoms both in men and women. The combined effect of smoking and occupational exposure was the most marked risk factor yielding the highest prevalence estimates. Thus, anti-smoking efforts at population level and measures to stop smoking and irritant exposure at work may reduce nasal symptoms. The campaign for Tobacco-Free Finland at 2030 [30] and the Healthy Workplaces Campaign [31] have been launched and should be strongly promoted.
